# *The Tree Is My Anchor*: A Pilot Study on the Treatment of BED through Nature-Based Therapy

**DOI:** 10.3390/ijerph15112486

**Published:** 2018-11-08

**Authors:** Sus Sola Corazon, Ulrik Sidenius, Katrine Schjødt Vammen, Sabine Elm Klinker, Ulrika Karlsson Stigsdotter, Dorthe Varning Poulsen

**Affiliations:** 1 Department of Geoscience and Natural Resource Management, Rolighedsvej 23, 1958 Frederiksberg C, Denmark; us@ign.ku.dk (U.S.); uks@ign.ku.dk (U.K.S.); dvp@ign.ku.dk (D.V.P.); 2 Danish Cancer Society, Strandboulevarden 49, 2100 Copenhagen Ø, Denmark; ksv@cancer.dk; 3 National Knowledge Center on Eating Disorders and Self Harm, Dronningens Tværgade 46, 1302 Copenhagen C, Denmark; kv@vioss.dk

**Keywords:** eating disorder, binge eating, therapy garden, health-promoting natural environments, acceptance and commitment therapy, health design, eating disorder examination, psychological general well-being

## Abstract

Binge eating disorder (BED), characterized by recurrent episodes of binge eating with a subjective experience of lack of control, is the world’s most common eating disorder. The aim of the present pilot study was to examine the feasibility of implementing nature-based therapy (NBT) in the treatment of BED. The NBT intervention was compared to Support Group Meetings (SGMs), which are the only publicly available form of support for people diagnosed with BED in Denmark. Twenty participants with a BED diagnosis were included in the study, which had a mixed-methods design including Eating Disorder Examination interviews, semi-structured interviews, and questionnaires measuring well-being (The Psychological General Well-Being Index) and self-esteem (Rosenberg’s Self-Esteem Scale). Both the NBT and the SGMs showed positive results on all outcome measures (decreases in binge eating episodes and increases in general psychological well-being and self-esteem). The interviews indicated that the NBT context made the psychotherapeutic content more accessible to the participants and further helped them transfer the therapeutic gains to daily life after completing treatment. However, these results should be interpreted with caution due to the small sample size—ideally, they would need to be tested on a larger, randomized sample.

## 1. Introduction

Binge eating disorder (BED) was included as an autonomous eating disorder in the American Diagnostic and Statistical Manual of Mental Disorders (DSM-5) in 2013 [1]. It is the world’s most common eating disorder and has been estimated to have a lifetime prevalence of 1.3–1.8 percent within different populations [2]. Since Denmark, where this pilot study is located, follows the World Health Organization’s International Classification of Diseases (ICD) as a diagnostic guide, BED is still not recognized as an autonomous eating disorder but is expected to become so this year [3]. BED is characterized by recurrent episodes of binge eating once a week or more over a period of several months, combined with a subjective experience of lack of control and the absence of compensatory behaviors, such as vomiting or laxative use [1]. BED is also associated with medical and psychiatric comorbidities such as obesity, diabetes, hypertension, and pain conditions [4], as well as depression and anxiety disorders [5].

Although it is estimated that approximately 40,000–50,000 Danes currently suffer from BED [6], there are no national publicly-available treatments in Denmark besides the weight control plans and consultations offered by health practitioners (covered by public health insurance) and free support group meetings organized by the National Association against Eating Disorders and Self Harm (LMSOS). Due to the current lack of treatment initiatives in Denmark, the Nature, Health & Design research group at the University of Copenhagen began a collaboration with the National Knowledge Center on Eating Disorders and Self Harm (VIOSS) in order to test the feasibility of implementing nature-based therapy (NBT) in the treatment of BED.

Since NBT is characterized by using bodily-engaging experiences and activities in nature as therapeutic tools [7,8], its physical approach to creating mental change seemed a potentially fruitful one. While the way psychotherapeutic NBT is conducted depends on the underlying therapeutic approach, NBT also has therapeutic potential on its own. One potential advantage intrinsic to NBT concerns bodily experiences through sensorimotor stimulation in the natural environment, which has been found to enhance mental and physical well-being [9,10]. Another concerns the mirroring process: being in a natural environment is demonstrably perceived as comforting by clients in NBT, and offers metaphors by which they can mirror themselves and frame their anxieties [8,11]. The NBT in the present project has so far been validated with regard to stress-related illnesses [12], but to the authors’ knowledge there is no current published research on the effect of NBT in the treatment of eating disorders.

In the present study, the psychotherapeutic approach in the NBT was based on Acceptance and Commitment Therapy (ACT), a cognitive psychotherapeutic approach, born out of Cognitive Behavioral Therapy (CBT), which draws on mindfulness-based therapy [13]. Along with other CBT-based interventions, it has been validated as an efficient treatment for BED [14,15]. It was chosen as the psychotherapeutic approach in this study as it specifically targets emotional and experiential avoidance [16], which are regarded as the primary maintaining mechanisms of binge eating [17] (for more information on the treatment, see Appendix A: NBT Treatment Manual).

The aim of the present study was to test the feasibility and potential of conducting BED treatment in an NBT context. Since it was designed as a comparative pilot study, one group of ten participated in NBT, and another group of ten participated in support group meetings (SGMs). The SGMs are offered by the National Association against Eating Disorders and Self Harm (LMSOS), which is currently Denmark’s only national freely-available program for people with BED. The main aim of the study was not to determine which intervention was better but to test whether an ACT-based NBT intervention could be a feasible treatment for BED by comparing it to the only national option available.

### 1.1. Research Questions

Did the NBT/SGMs result in reduced episodes of binge eating and remission of aspects of the psychopathology?

Did the NBT/SGMs result in increased self-esteem and increased psychological general well-being?

### 1.2. Research Question Specifically for the NBT

How was being in nature and doing nature-based activities experienced by the participants in relation to their therapeutic process?

How were the therapeutic gains implemented in the participants’ daily lives after ending the therapy?

## 2. Materials and Methods

### 2.1. Recruitment

The participants were recruited through the online newsletters of LMSOS. Recruitment closed when ten of the newsletter readers had signed up to participate in the NBT or the SGMs, and the remainder were placed on a waiting list. To be eligible for inclusion, the participants had to meet the diagnostic requirement for BED according to the DSM-5 [1], while exclusion criteria were severe psychiatric morbidity, psychotic disorders, personality disorders, suicidal tendencies, and drug or alcohol abuse. The initial screening was performed by a psychologist at LMSOS followed by an Eating Disorder Examination (EDE) interview performed by a staff member at VIOSS trained in the EDE interview method [18]. All participants who had signed up met the inclusion criteria both in the NBT and SGMs group. Ten participants were enrolled in the SGMs group, and ten participants in the NBT group. After the first session with the SGMs group, one participant dropped out citing the difference in age from the other participants: a new participant was therefore recruited from the waiting list and put through the screening process before enrolment.

The participants were fully informed about the aim, content, and purpose of the study before signing their consent to participate, in accordance with the guidelines provided by the National Committee on Health Research Ethics. The study was approved by the Danish Data Protection Agency (J.nr.: 519-0070/17-5000). The study was also presented to The National Committee on Health Research Ethics to establish whether approval would be required. The Committee determined that no approval was needed, due to the nature of the study.

### 2.2. Interventions

#### 2.2.1. Nature-Based Therapy

The NBT consisted of one session of three hours per week for a duration of 12 weeks, starting in the early autumn and finishing in the beginning of winter. The sessions were held in Nacadia^®^, the University of Copenhagen’s 1.4-hectare forest therapy garden. It is located in an arboretum, which contains a unique collection of approximately 2000 different trees and shrubs and appears as a beautiful park—or exotic forest. The therapy garden is designed in accordance with the model for evidence-based health design [19]. The garden is enclosed by a living fence of trees and shrubs to evoke feelings of safety and serenity, and it includes a variety of natural qualities designed to promote psychological and physical restoration: these include a brook, small secluded retreats surrounded by vegetation, and more open areas including a meadow for group activities.

The concept for the specific NBT was originally developed by Corazon and colleagues [7], and later adjusted to accommodate different user groups such as veterans with post-traumatic stress disorder [8]. It puts physical presence and activities in nature center-stage in the therapeutic process. The NBT for the participants with BED involved a variety of guided body and mind awareness exercises in the natural environment. These included seated awareness exercises around a bonfire (integrating stimuli such as the scents, sounds, and heat from the fire), walking awareness exercises in the garden, relaxation exercises such as body-scanning (either lying on the grass or sitting in the garden), and stretching exercises in the meadow. The participants’ increased physical and mental awareness formed the basis for therapeutic work highlighting the connection between body, emotions, thoughts, and patterns of action in accordance with the core processes in ACT: being present, acceptance, cognitive defusion, self as context, values, and committed action [20]. While the exercises in ACT are usually performed indoors using mental imaging and metaphors, in NBT one can bring metaphors to physical life in an outdoor setting. Thus, if changing thoughts are represented by an image of leaves falling into a stream, then the physical dropping of real leaves into an actual stream will make the mental exercise more tangible and will improve its quality as a memory [8].

Each session in the NBT followed the same structure and had a specific theme in accordance with the core processes in ACT. Two therapists facilitated the therapy. One was a licensed psychologist with training in ACT and the other a licensed psychotherapist, mindfulness, and yoga instructor. The treatment program is described in detail in Appendix A: NBT treatment manual.

#### 2.2.2. Support Group Meetings

The support group meetings (SGMs) consisted of a 10-week run with one weekly three-hour session held at the headquarters of LMSOS, which corresponds to the usual structure and duration of SGMs offered at LMSOS. The meetings were facilitated by a licensed psychologist, and their objective was to use social support as therapeutic means for change. In the first session, each group member decided on a personal focus to work with throughout the 10 weeks. The subsequent sessions all followed the same structure: one group member shared his or her thoughts, feelings, hopes, or goals, and the other group members reflected on what had been said. The meetings were not presented as psychotherapy but as a social support tool to boost self-esteem and diminish the symptoms of the eating disorder.

### 2.3. Outcome Measures

The study had a mixed-methods design, making use of both quantitative and qualitative approaches in the methodology [21]. This path was chosen in order to examine the effect of the two interventions and to gain insight into what the participants experienced in and gained from the NBT.

#### 2.3.1. Primary Outcome Measure: Eating Disorder Examination Interview

The participants underwent an Eating Disorder Examination (EDE) interview before and after the interventions: this EDE served both as a diagnostic tool for inclusion and as an outcome measure. The EDE interview was semi-structured and entailed questions concerning the frequency of behaviors and attitudes indicative of an eating disorder [22]. The questions mainly focused on the preceding 28 days, but some covered the preceding three months. The answers were rated on a seven-point scale from 0–6, with a score of zero indicating that the feature in question was not present. The severity of various aspects of the psychopathology were rated in four sub-scales: restraint, eating concern, shape concern, and weight concern, as well as a global score. The EDE interview has been validated through previous research [23].

#### 2.3.2. Secondary Outcome Measures: Questionnaires

The participants were sent an e-mail invitation to fill out a web-based three-part questionnaire before and after the interventions. The first part entailed questions concerning age, gender, and personal history related to BED (year BED started, comorbidities, and previous treatment).

The second part of the questionnaire, Rosenberg’s Self-Esteem Scale (RSES), measured the participants’ sense of self-esteem and included 10 questions rated on a four-point scale from 0–3 (five of the questions had reverse scoring). The total score ranged from 0–30 in which scores below 15 suggested low self-esteem [24]. The questionnaire has been validated through previous research [25].

The third part, the Psychological General Well-Being Index (PGWBI) questionnaire, measured six dimensions of psychological general well-being: anxiety, depressed mood, positive well-being, self-control, general mental health, and vitality, as well as a global score. The 22 questions were rated on a six-point scale from 0–5, with a highest possible global score of 110. It is the most widely used patient outcome measure, and its reliability and validity have been confirmed through previous research [26].

### 2.4. Interviews

Three months after the conclusion of the NBT intervention, the participants received an email invitation to participate in a telephone interview with the objective to explore what the participants had gained from the NBT, and whether they had subsequently implemented these gains into their daily lives. Semi-structured interviews, lasting 25 min on average, were held with the four participants who agreed to participate. They were recorded with the participants’ consent and later transcribed for additional analysis. The interview guide and questions are entailed in Appendix B: Interview guide.

Based on a content analysis [27], the following procedure was used. First, the recordings of the interviews were reviewed several times. Then the transcripts were read, and sections of interest (meaning units) were marked. Finally, the meaning units from all interviews were collected, placed in a matrix, and analyzed for shared and individual content.

### 2.5. Statistics

Differences between baseline and endpoint scores were estimated with a Wilcoxon signed rank test for paired data. The significance level was set at *p* < 0.05 in a two-tailed test. Effect sizes were calculated as Pearson’s *r* for samples not showing normal distribution, and the data was analyzed per protocol, including only those individuals who completed the interventions. The statistician performing the analyses was not blinded concerning the specific treatment received.

## 3. Results

### 3.1. Participants’ Characteristics and Dropouts

Eight of the participants in the NBT group (eight women) and seven participants in the SGMs group (six women and one man) completed their respective intervention. Two participants dropped out of the NBT group citing lack of time due to personal circumstances, and three dropped out of the SGMs group. Of these, one participant was ‘not feeling ready for therapy’ and another was ‘not comfortable with the group format’; the reason behind the third dropout was not known. The mean age of the completing participants was 47 years of age in the NBT group and 41 years of age in the SGMs group. Generally, the participants stated that their eating disorder started at a young age (NBT mean: 13 years of age; SGMs mean: 19 years of age). Four participants in each group stated that they also suffered from other illnesses.

### 3.2. EDE Interviews

The results from the EDE interviews showed that the participants still fulfilled the criteria for BED at the end of both interventions (Table 1). However, the mean binge-eating episodes were significantly diminished in the NBT group after the intervention (*p* < 0.05), and the effect size indicated a medium effect (*r* > 0.50) [28]. According to the severity grading in DSM-5, the mean scores for both the NBT and SGMs groups were in the high end of the ‘mild’ spectrum before the respective interventions started. After the NBT, the mean binge-eating episodes were reduced to the very low end of the ‘mild’ spectrum. This was not the case for the SGMs group.

The mean scores concerning the severity of aspects related to the psychopathology were diminished in all scales in both the NBT and the SGMs groups, except ‘Weight concern’ in the SGMs group (Table 2). These, however, were not found to be significant.

### 3.3. Rosenberg’s Self-Esteem Scale

The results from the Rosenberg Self-Esteem Scale (RSES) showed an increase in mean score for both the NBT and the SGMs group after their respective interventions (Table 3), although the change was only significant for the NBT group (*p* < 0.05). The effect sizes indicated a medium effect for the NBT (*r* > 0.50) and no effect for the SGMs (*r* < 0.20).

### 3.4. Psychological General Well-Being Index

The scores on the Psychological General Well-Being Index showed a significant increase in mean value for both the NBT group (*p* = 0.05) and the SGMs group (*p* < 0.05). The mean score for the NBT group after the intervention approached the mean value for a healthy Danish sample (73.14) provided by the MAPI Research Institute [25].

### 3.5. Interviews

The content analysis of the interviews provided insight into how the interviewees experienced and benefited from participating in the NBT. The results of the analysis are presented below in relation to the contents of interest.

#### 3.5.1. The Experience of Natural Surroundings and Nature-Based Activities in Relation to the Therapeutic Process

The interviewees agreed that they experienced the therapy garden as a safe and protective framework for the therapy. They described it as “*calming*”, “*supportive*”, “*protective*”, “*motivating*”, “*a feeling of refuge*”, and “*providing mental space*”. They also described how being in a natural environment had helped them become more grounded and present: “*You calmed down and entered into this meditative state, where you were able to process everything better*”.

The awareness exercises that integrated sensory experiences and movement made the therapeutic content more accessible to the interviewees: “*The way the exercises are conducted made them more understandable and accessible*”, said one participant. Another reported that: “*the message penetrated more deeply because you were more present*”. A third reflected on the difference between undergoing psychotherapy indoors and outdoors:

“*When you do awareness exercises in nature, you experience the sunshine, the smell of the grass, you feel the ground, so you are not caught in the swarm of your own thoughts. The surroundings carry you with them, you are in the world and not in a confined room, it is much closer to being in the real world.*”

All the interviewees explained how the use of metaphors relating to the natural environment “*made things more relatable to oneself*” and shared recollections of exercises such as mirroring oneself in a tree with solid roots in the ground: “*It was very good to relate to—a metaphor I definitely can lean on*”. Some developed personal relationships with natural objects. For example, two interviewees explained how a tree served as a reference point for them throughout the therapy period: “*I developed a relationship with the tree. It was my anchor*”; “*The tree gave me some steering […] it made the whole period more accessible for me*.” Another participant described how she could see herself reflected in a pine cone during an exercise about self-nurturing.

In general, the integration of the natural environment into the exercises was experienced as motivating and as adding more holistic or grounded dimensions to the mental work, as summarized by this participant: “*It’s a step beyond being stuck in your own head all the time*”.

#### 3.5.2. Benefits from NBT and Implementation into Daily Life

The interviewees shared stories of how they continued to benefit from the NBT after the intervention because they had developed tools they could use to cope with emotional stress.

“*The awareness exercises help me calm down by just sitting observing a tree, bending in the wind*”; “*I have become aware of using sensory experiences around me as a way to keep from being overwhelmed by feelings. To stop for a moment and, for example, replay the exercise by observing the details of the environment around me*.”

“*I might find myself on the bus, overwhelmed by destructive thoughts and emotions. Then I’ll look out of the window and say, ‘I see a tree now,’ and then I spot a bird in it, and it’s just like being back in the therapy garden*”.

The acquired awareness tools also changed the way some of the interviewees interacted with nature in their everyday lives. For example, one interviewee recounted how the intervention had added a new dimension to her daily walk near a local lake: “*I am consciously aiming at being present in the current moment during my walk. Rather than shutting out the world and being caught up in my thoughts and emotions, I try to be present in the world in which I am, so I see the surroundings and feel the ground beneath my feet*”.

Some exercises had a key impact on individual interviewees. One used a natural object to which she particularly related in one of the exercises for support in her daily life; “*The pine cone is always within easy reach because I used it a lot during the self-nurturing exercise. I still give that a lot of thought*”. Another shared how a physical exercise in the meadow led to an understanding she could apply to reducing binge eating: “*It will hurt along the way, but it’s a part of becoming better in the end*”.

## 4. Discussion

Both interventions showed positive results according to all outcome measures (reduced binge eating episodes, increased psychological general well-being, and increased self-esteem), though not all proved significant. The effect sizes were larger for all outcome measures for the NBT compared with the SGMs. However, the small sample size should be taken into account when interpreting the results, as it made the statistical analysis vulnerable to the influence of extreme cases, and probably resulted in the overall significance of the results being quite low. The results should therefore mainly be seen as indications.

The therapeutic mechanisms at play in outdoor psychotherapy are far from fully understood. With that said, theory development and related research is starting to emerge (e.g., [8,11]) and the results from the current study support integrating physical exercises in the natural environment as a means of making therapy more accessible both during the intervention and in everyday life. The interviewees’ accounts of mirroring and seeking support in natural objects reinforce the idea that the natural environment can act as a mirror or ‘second therapist,’ as proposed by Jordan [11]. The interviews also showed that the participants continued to deploy the NBT awareness exercises to deal with emotional difficulties. This indicates that NBT holds an interesting potential with regard to its ability to sustain a long-term effect of the therapy, as research shows that lack of remission in individuals with BED after psychotherapeutic treatment is linked to the emotional discomfort associated with implementing the learned strategies [16]. However, the very small number of interviewees should be taken into account when generalizing the results. Also, only four participants agreed to participate in the interviews, which might have been the ones who had benefitted the most from the NBT.

The intervention did not include diet or focus on weight loss. This could be seen as a deficiency in the present study, as binge eating disorder is highly related to obesity [29], which is also linked with other serious physical co-morbidities [5] and increased health care costs [30]. However, other CBT-based interventions, which addressed diet and weight loss, did not find any significant effect on weight loss [4]. Also, there is a concern about including weight loss in BED treatment because the psychopathology is sustained by increased focus on eating, weight, and shape [22], therefore emphasizing dieting could reinforce the psychopathology. The question is therefore how to include these aspects in a NBT treatment.

A possible approach might be to include nature activities focused on food gathering in the natural environment, alongside preparing and eating food during the group therapy sessions. This departure from the traditional approach of providing information on diet and nutrition would ensure a more ‘holistic’ approach to food intake.

### Limitations

The study has several limitations. Firstly, the sample is very small and non-randomized, and the results can therefore only be read as indications. The non-randomization risks a placebo effect because if the participants are particularly motivated about a chosen intervention, they will expect and indeed perhaps create a positive outcome.

Secondly, there is an overrepresentation of women. This, however, could be explained by figures estimating that two-thirds of the individuals suffering from BED in Denmark are female [6], and that women are more likely than men to seek treatment for eating disorders [2].

A third limitation, general to the field of NBT and also the present study, is the difficulty in determining to what extent the result stems from the NBT, and to what extent from the psychotherapeutic approach. While it is impossible to separate the two in an actual intervention, the problem could be addressed by having a control group that only receives the psychotherapy in its traditional indoor form—in this case, ACT. Therefore, the study design itself, with a control group receiving SGMs, also proves to be a limitation when it comes to interpreting the results. It is therefore advised that a future study design in the field of integrating NBT in the treatment of BED uses randomization and a control group receiving the same psychotherapeutic intervention without the use of nature-based activities and experiences as therapeutic tools.

## 5. Conclusions

The results provide initial support for the feasibility of implementing an ACT-based NBT in the treatment of BED. One of the potentials of NBT seems to be that it helps concretizing and ‘anchoring’ the therapeutic content through physical experiences and exercises, thereby making it more accessible and applicable for the participants. However, the results need to be tested in a larger randomized sample. In addition, it is advised that diet and weight loss should be included in a future intervention.

## Figures and Tables

**Table 1:** Fulfilment of criteria for binge eating disorder (BED) and frequency of binge-eating behavior before and after the interventions. NBT: nature-based therapy; SGMs: Support Group Meetings.

NBT (*N* = 8)					SGMs (*N* = 7)			
Outcome Variable	Before Mean (SD)	After Mean (SD)	Significance *p*	Effect Size *r*	Before Mean (SD)	After Mean (SD)	Significance *p*	Effect Size *r*
Fulfil the criteria for BED	8	7	>0.05	<20	7	7	>0.05	<20
Binge-eating episodes within the last month	21.5 (29.56)	3.5 (4.38)	0.041	0.54	13.7 (10.31)	10.9 (8.97)	0.611	0.14

SD: Standard Deviation

**Table 2:** Scores on Eating Disorder Examination (EDE) scales concerning the severity of aspects of psychopathology before and after intervention.

	NBT (*N* = 8)		(SGMs *N* = 7)	
EDE Scales	Before Mean	After Mean	Before Mean	After Mean
Restraint	2.3	1.7	2.9	2.3
Eating concern	2.7	1.8	2.7	2.0
Shape concern	4.2	3.4	4.7	3.9
Weight concern	4.4	2.6	3.9	4.5
Global score	3.4	2.4	3.6	3.2

Note: The difference in score before and after the interventions was not significant for any of the scales (*p* > 0.05).

**Table 3:** Scores on Rosenberg Self-Esteem (RSES) and Psychological General Well-Being Index (PGWBI) before and after intervention.

NBT (*N* = 8)					SGM (*N* = 7)			
Outcome Variable	Before Mean (SD)	After Mean (SD)	Significance *p*	Effect Size *r*	Before Mean (SD)	After Mean (SD)	Significance *p*	Effect Size *r*
RSS	14.63 (7.05)	19.86 (6.81)	0.018	0.59	10.71 (7.06)	14.57 (7.45)	0.549	0.16
PGWBI (global score)	51.63 (17.62)	67.75 (13.20)	0.050	0.49	51.86 (24.40)	60.00 (26.75)	0.468	0.23

## References

[1] American Psychiatric Association (2013). Diagnostic and Statistical Manual of Mental Disorder.

[2] Kessler R.C., Berglund P.A., Chiu W.T., Deitz A.C., Hudson J.I., Shahly V., Aguilar-Gaxiola S., Alonso J., Angermeyer M.C., Benjet C. (2013). The prevalence and correlates of binge eating disorder in the WHO World Mental Health Surveys. Biol. Psychiatry.

[3] World Health Organization ICD-11 Betadraft. https://icd.who.int/dev11/l-m/en#/http%3a%2f%2fid.who.int%2ficd%2fentity%2f1673294767.

[4] Olguin P., Fuentes M., Gabler G., Guerdjikova A.I., Keck P.E., McElroy S.L. (2017). Medical co-morbidity of binge eating disorder. Eat. Weight Disord..

[5] Grilo C.M., White M.A., Masheb R.M. (2009). DSM-IV psychiatric disorder comorbidity and its correlates in binge eating disorder. Int. J. Eat. Disord..

[6] National Association against Eating Disorders and Self Harm. https://www.lmsos.dk/viden/spiseforstyrrelser/?gclid=Cj0KCQjw3KzdBRDWARIsAIJ8TMQkYaQTEOXERLCkA6pMrylpZk6xcE_Hjx0MG0woY52FoIwHP_lMEw0aAlGIEALw_wcB.

[7] Corazon S.S., Stigsdotter U.K., Jensen A.G., Nilsson K. (2010). Development of the nature-based therapy concept for patients with stress-related illness at the Danish healing forest garden Nacadia. J. Ther. Hortic..

[8] Corazon S.S., Schilhab T., Stigsdotter U.K. (2010). Developing the therapeutic potential of embodied cognition and metaphors in nature-based therapy: Lessons from theory to practice. J. Adv. Educ. Outdoor Learn..

[9] Poulsen D.V., Stigsdotter U.K., Djernis D., Sidenius U. (2016). “Everything just seems much more right in nature”: How veterans with post-traumatic stress disorder experience nature-based activities in a forest therapy garden. Health Psychol. Open.

[10] Sidenius U., Stigsdotter U.K., Poulsen D.V., Bondas T. (2017). “I look at my own forest and fields in a different way”: The lived experience of nature-based therapy in a therapy garden when suffering from stress-related illness. Int. J. Qual. Stud. Health Well-Being.

[11] Jordan M., Jordan M., Hinds J. (2016). Ecotherapy as psychotherapy: Towards an ecopsychotherapy. Ecotherapy: Theory, Research and Practice.

[12] Stigsdotter U.K., Corazon S.S., Sidenius U., Larsen H.B., Fjorback L. (2018). Efficacy of nature-based therapy for individuals with stress-related illnesses—A randomized controlled trial. Br. J. Psychiatry.

[13] Baer R.A., Krietemeyer J., Baer R.A. (2005). Overview of mindfulness- and acceptance-based treatment approaches. Mindfulness-Based Treatment Approaches. Clinician’s Guide to Evidence Base and Application.

[14] Manlick C.F., Cochran S.V., Koon J. (2013). Acceptance and commitment therapy for eating disorders: Rationale and literature review. J. Contemp. Psychother..

[15] Palvras M.A., Hay P., Filho C.A.S., Claudino A. (2017). The efficacy of psychological therapies in reducing weight and binge eating in people with bulimia nervosa and binge eating disorder who are overweight or obese—A critical synthesis and meta-analyses. Nutrients.

[16] Juarascio A.S., Manasse S.M., Espel H., Forman E.M. (2017). Developing an acceptance-based behavioral treatment for binge eating disorder: Rationale and challenges. Cogn. Behav. Pract..

[17] Grilo C.M., Fairburn C.G., Brownell K.D. (2002). Binge eating disorder. Eating Disorders and Obesity: A Comprehensive Handbook.

[18] CREDO Eating Disorder Examination. http://credo-oxford.com/pdfs/EDE_17.0D.pdf.

[19] Stigsdotter U.K. (2015). Nature, health & design. CIPRA.

[20] Hayes S.C., Luoma J.B., Bond F.W., Masuda A., Lillis J. (2006). Acceptance and commitment therapy: Model, processes and outcomes. Behav. Res. Ther..

[21] Creswell J.W., Wicky L., Clark P. (2010). Designing and Conducting Mixed Methods Research.

[22] Fairburn C.G., Cooper Z., Shafran R. (2002). Cognitive behavior therapy for eating disorders: A “transdiagnostic” theory and treatment. Behav. Res. Ther..

[23] Fairburn C.G., Cooper Z., Fairburn C.G., Wilson T.G. (1993). The eating disorder examination. Binge Eating: Nature, Assessment and Treatment.

[24] Blascovich J., Tomaka J., Robinson J.P., Shaver P.R., Wrightsman L.S. (1993). Measures of self-esteem. Measures of Personality and Social Psychological Attitudes.

[25] Robins R.W., Hendin H.M., Trzesniewski K.H. (2001). Measuring global self-esteem: Construct validation of a single-item measure and the Rosenberg Self-Esteem Scale. Pers. Soc. Psychol. Bull..

[26] Chassany O., Diderot E., Dimenäs D., Wu A., Dupuy H. (2004). The Psychological General Well-Being Index: User Manual.

[27] Shannon S.E., Hsieh H.F. (2005). Three approaches to qualitative content analysis. Qual. Health Res..

[28] Cohen J. (1992). A power primer. Psychol. Bull..

[29] Amianto F., Lavagnino L., Abbate-Daga G., Fassino S. (2011). The forgotten psychosocial dimension of the obesity epidemic. Lancet.

[30] Tremmel M., Gerdtham U.G., Nilsson P.M., Saha S. (2017). Economic burden of obesity: A systematic literature review. Int. J. Environ. Res. Public Health.

